# Supplementary material for: bigPint: A Bioconductor visualization package that makes big data pint-sized
Source: PLoS Comput Biol. 2020 Jun 15;16(6):e1007912. doi: 10.1371/journal.pcbi.1007912 (PMC7347224; doi:10.1371/journal.pcbi.1007912)
Supplement: S1 Table — (PDF) [file pcbi.1007912.s001.pdf]

S1 Table. Resources for developers about bigPint interactive graphics

Plot	Pseudocode	Code
Scatterplot matrix	S1 Pseudocode	bit.ly/spmCode
Litre plot	S2 Pseudocode	bit.ly/litreCode
Volcano plot	S3 Pseudocode	bit.ly/volcCode
Parallel coordinate	S4 Pseudocode	bit.ly/pcpCode

Note: The pseudocode and code provided in supplementary material is intended for *developers* who wish to understand the details of how we created the double-layered interactivity and possibly repurpose and modify these fairly new technology for new software. Biology researchers who wish to use the bigPint package should instead consult the bigPint package website (https://lindsayrutter.github.io/bigPint) and the associated manuscripts.
