# Supplementary material for: bigPint: A Bioconductor visualization package that makes big data pint-sized
Source: PLoS Comput Biol. 2020 Jun 15;16(6):e1007912. doi: 10.1371/journal.pcbi.1007912 (PMC7347224; doi:10.1371/journal.pcbi.1007912)
Supplement: S1 Pseudocode — (PDF) [file pcbi.1007912.s002.pdf]

Data: Data frame input by user
Result: Interactive scatterplot matrix

```
/* Declare Shiny server
server ← function(input, output, session){
    /* Declare Shiny output scatterplot matrix
    output$scatMatPlot ← renderPlotly({
        /* Draw hexagons and x=y line in bottom-left corner of matrix
        my_fn ← function(data, mapping){}
        /* Create static scatterplot matrix
        p ← ggpairs(data, lower = list(continuous = my_fn))
        /* Convert ggplot2::ggplot() object to plotly object
        ggP ← ggplotly(p)
        /* Tailor plotly scatterplot matrix interactivity with JavaScript
        ggPR ← ggP %>% onRender("function(el, x, data){
            /* If the user clicks on the plotly scatterplot matrix object
            el.on('plotly_click', function(e){
                /* Delete any old superimposed plotly geoms (orange dots)
                if (x.data.length > 0){Plotly.deleteTraces(el.id)}
                /* Determine gene IDs selected by user click. Save as object called selID with handle called 'selID' so it
                  can be read outside current JavaScript function back in Shiny
                Shiny.onInputChange('selID', selID)
                /* Create traces for selected gene IDs as orange points that state gene names upon hovering
                trace = {mode: 'markers', color: 'orange', size: 6, text: selID, hoverinfo: 'text'}
                /* Superimpose traces onto the plotly scatterplot matrix object
                Plotly.addTraces(el.id, Traces)
            })
        })
        /* Pass the R data object into the JavaScript function
        ", data = data
    })
    /* Read into Shiny the gene IDs that user clicked on
    selID ← reactive(input$selID)
    /* Create data subset (read counts) for only the selected gene IDs
    pcpDat ← reactive(data[which(data$ID %>% selID()), ])
    /* Create static box plot of the full dataset
    BP ← ggplot(data) + geom_boxplot()
    /* Render boxplot interactive as a plotly object
    ggBP ← ggplotly(BP)
    /* Declare Shiny output boxplot
    output$boxPlot ← renderPlotly({
        /* Tailor interactivity of the plotly boxplot object using custom JavaScript
        ggBP %>% onRender("function(el, x, data){
        /* Create traces for selected gene IDs as orange lines that state gene names upon hovering
        trace = {mode: 'lines', color: 'orange', width: 1.5, text: selID, hoverinfo: 'text'}
        /* Push traces to be superimposed onto the plotly scatterplot matrix object
        Plotly.addTraces(el.id, Traces)
        /* Pass R objects into the JavaScript function
        }", data = list(pcpDat = pcpDat())
    })
}
```

S1 Pseudocode: Pseudocode for interactive scatterplot matrix
