# Supplementary material for: bigPint: A Bioconductor visualization package that makes big data pint-sized
Source: PLoS Comput Biol. 2020 Jun 15;16(6):e1007912. doi: 10.1371/journal.pcbi.1007912 (PMC7347224; doi:10.1371/journal.pcbi.1007912)
Supplement: S3 Pseudocode — (PDF) [file pcbi.1007912.s004.pdf]

Data: Data frame input by user
Result: Interactive volcano plot

```
/* Declare Shiny server
server ← function(input, output, session){
    /* Shiny user input options
    /* Define largest fold change dynamically based on data
    fcInMax ← max(ldply(dataMetrics, rbind)[["logFC"]])
    /* Construct dynamic input Shiny slider for fold change
    output$slider ← renderUI(sliderInput("logFC", "Log fold change:", min=0, max=fcInMax, step=0.1))
    /* Declare shiny output volcano plot
    output$volPlot ← renderPlotly({
        /* Create reactive expression of plotly background volcano plot
        gP ← reactive(p ← ggplot(data); gP ← ggplotly(p))
        /* Create reactive expression of plotly background volcano plot
        plotlyVol ← reactive(gP())
        /* Tailor interactivity of the plotly volcano plot object using custom JavaScript
        plotlyVol() %>% onRender("function(el, x, data){
            /* Read handle called 'points' to obtain variables sent from R into JavaScript
            Shiny.addCustomMessageHandler('points', function(drawPoints){
                /* Delete any old superimposed plotly geoms (dots)
                if (x.data.length > 0){Plotly.deleteTraces(el.id)}
                /* Create traces for selected gene IDs as points that state gene names upon hovering
                trace = {x: drawPoints.geneX, y: drawPoints.geneY, mode: 'markers', color: drawPoints.pointColor, size:
                  drawPoints.pointSize, text: drawPoints.geneID, hoverinfo: 'text'}
                /* Superimpose traces onto the plotly volcano plot object
                Plotly.addTraces(el.id, trace)
            })
        }")
    })
    /* If the user changes their input, store information about new superimposed genes with a handle called 'points'.
      These values can then be sent from R to JavaScript
    observe({session$sendCustomMessage(type = "points", message=list(geneX=geneX, geneY=geneY, pointSize =
      pointSize, geneID=geneID, pointColor=pointColor))})
    /* Declare Shiny output boxplot
    output$boxPlot ← renderPlotly({
        /* Create reactive expression of plotly background boxplot
        BP ← reactive(ggplot() + geom_boxplot())
        ggBP ← reactive(ggplotly(BP()))
        /* Tailor interactivity of the plotly boxplot object using custom JavaScript
        ggBP() %>% onRender("function(el, x, data){
            /* Read handle called 'lines' to obtain variables sent from R into JavaScript
            Shiny.addCustomMessageHandler('lines', function(drawLines){
                /* Delete any old superimposed plotly geoms (lines)
                Plotly.deleteTraces(el.id, traceLine)
                /* Create xArr and yArr, array of x and y values for superimposed lines, from drawLines
                /* Create traces for selected gene IDs as lines that state gene names upon hovering
                traceLine = {x: xArr, y: yArr, mode: 'lines', color: drawLines.pointColor, width: 2, opacity: 0.9; text:
                  drawLines.geneID, hoverinfo: 'text'}
                /* Superimpose traces onto the plotly volcano plot object
                Plotly.addTraces(el.id, traceLine)
            })
        })
    })
    /* If the user changes their input, store information about new superimposed genes with a handle called 'lines'.
      These values can then be sent from R to JavaScript
    observe({session$sendCustomMessage(type = "lines", message=list(geneID=geneID, pointColor=pointColor))})
}
```

S3 Pseudocode: Pseudocode for interactive volcano plot
