# Supplementary material for: bigPint: A Bioconductor visualization package that makes big data pint-sized
Source: PLoS Comput Biol. 2020 Jun 15;16(6):e1007912. doi: 10.1371/journal.pcbi.1007912 (PMC7347224; doi:10.1371/journal.pcbi.1007912)
Supplement: S4 Pseudocode — (PDF) [file pcbi.1007912.s005.pdf]

```
Data: Data frame input by user
Result: Interactive parallel coordinate plot
/* Declare Shiny server
server ← function(input, output, session){
    /* Create an empty ggplotly object with same dimensions as data
    p ← ggplot(); gp ← ggplotly(p)
    /* Send user-defined line color into onRender() function
    session$sendCustomMessage(type = "lines", message = list(lineColor = lineColor))
    /* Declare Shiny output parallel coordinate plot
    output$pcpPlot ← renderPlotly({
        /* Tailor interactivity of plotly parallel coordinate plot using custom JavaScript
        gp %>% onRender("function(el, x, data){
            /* Define rects array object to hold IDs of remaining lines
            var rects = []
            /* Draw lines for the original dataset
            var pcpLine = {x: xArr, y: yArr, mode: 'lines', color: data.lineColor}
            Traces.push(pcpLine)
            Plotly.addTraces(el.id, Traces)
            /* If the user draws a rectangle
            el.on('plotly_selected', function(e){
                /* Delete lines outside the user-defined rectangle
                Traces.push(delLine)
                Plotly.addTraces(el.id, Traces)
                /* Create new object called pcpDat that only contains genes within rectangle
                /* Push IDs of remaining lines to rects array object
                for (a=0; a<pcpDat.length; a++){rects.push(pcpDat[a]['ID'])}
                /* Save remaining gene IDs in an object called rects with handle called 'rects' so it can be read outside
                  current JavaScript function back in Shiny
                Shiny.onInputChange('rects', rects)
            })
        })
        /* Pass R objects into the JavaScript function
        ", data = list(pcpDat = pcpDat, lineColor = lineColor)
    })
    /* Read into Shiny the gene IDs that remain within rectangle drawn by user
    inputRectDf ← reactive(input$rects)
    /* Print the selected gene IDs in interactive data table
    output$rectdf = renderDataTable(pcpDat %>% filter(ID %in% inputRectDf()))
}
```

**S4 Pseudocode:** Pseudocode for interactive parallel coordinate plot
