# Supplementary material for: bigPint: A Bioconductor visualization package that makes big data pint-sized
Source: PLoS Comput Biol. 2020 Jun 15;16(6):e1007912. doi: 10.1371/journal.pcbi.1007912 (PMC7347224; doi:10.1371/journal.pcbi.1007912)
Supplement: Attachment — Submitted filename: ResponseToReviewers.doc [file pcbi.1007912.s007.doc]

We would like to thank the reviewers for their helpful feedback. Both reviewers brought up thoughtful suggestions, many of which we were able to successfully incorporate. We believe these updates will enhance software usability and integration with Bioconductor.

**Reviewer #1:**

1. The authors claim that their software can be used for the exploration of any large biological datasets. However, their choice of plots, in particular the volcanoplot, suggests to me a focus on RNA-seq data. I would suggest the inclusion of another example for a non RNA-seq dataset or making it clear that this package targets RNA-seq data exclusively.

With the exception of the volcano plot, most of these plots (scatterplot matrices, parallel coordinate plot, and litre plots) can broadly help users compare variability within and between treatment groups, a common theme in statistics. While this theme can likely be applied outside RNA-sequencing studies, we agree that all of our examples and main anticipation was to develop this package for RNA-sequencing analysis. We do not want users to feel misled into expecting examples outside of RNA-sequencing, so we have made several changes to wordings. Some examples are below:

a) We changed “We recently developed bigPint, an interactive data visualization software package available on Bioconductor. The bigPint package allows users to visually explore many types of large multivariate datasets, even though it was more specifically developed for RNA-seq data.” to “We recently developed bigPint, an interactive RNA-sequencing data visualization software package available on Bioconductor.”

b) We removed sentences that stated “If a researcher is using the package to visualize RNA-seq data, …”. This sentence format originally occurred twice in the “Basic input” subsection.

2. It is great that all plots are easily downloadable, but for the purpose of replicability it would be great if logs of all user interactions could be provided. This can, for example, be handled with shinylogs (https://github.com/dreamRs/shinylogs).

Thank you for the suggestion. It is possible that certain information cannot easily be stored in human-readable formats in shinylogs with our applications due to their large deviation from only built-in Shiny code and their tailored custom messages that transfer objects in different formats between plotly, htmlwidgets, shiny, etc. (as described in the fourth paragraph of the section “Independent layers of interactivity” in our paper). However, we agree it is a good idea to include shinylogs to provide as much information as possible in log format for users. We added an automatic shinylogs functionality to each of the interactive applications (litre plot, parallel coordinate plot, scatterplot matrix plot, and volcano plot). In each case, a JSON file with shinylogs output is saved to the temporary directory. A message is printed the console when the user closes the application that indicates that shinylogs output has been saved to the temporary directory.

3. Part of why I think the package feels clunky is that each plot is provided in a separate application. I think it would be more user friendly to provide one app that has user-specified outputs. A brilliant example of this is the iSEE package.

Thank you for this comment. Even though both iSEE and bigPint provide visualization methods, their background technology may be quite different. Our technology introduces fairly new double-layer interactivity that requires tailored custom messages that transfer objects in different formats between plotly, htmlwidgets, shiny, etc. (as described in the fourth paragraph of the section “Independent layers of interactivity” in our paper). We needed to deviate significantly from using only Shiny code. Because of the background technology, as it stands currently, it is difficult to provide a similar layout as iSEE (with all graphics in one Shiny application) without seeing significant drops in speed and reactivity.

Most plots in our package require similar user inputs (data AND dataMetrics object OR SummarizedExperiment object). Users can choose which input format they prefer. After that, users can follow a straightforward pipeline written in reproducible code on our website (https://lindsayrutter.github.io/bigPint/articles/pipeline.html). They can use their data or sample datasets provided with the package. We envision that even if users need to run code to produce different plots of interest, that they can do so in a streamlined fashion given our reproducible pipelines, sample datasets, help manuals with additional reproducible code, and the fact that each plotting tool requires the same input data formats.

4. Finally, I am unsure why the author chose the dataMetrics object as input, instead of the much more common SummarizedExperiment object. This has the advantage that it offers support for DelayedMatrices which are extremely useful when datasets are very large.

Thank you for sharing this suggestion. Our original thinking was to provide the most broadly-used R data structures for bigPint users. We thought this would increase the accessibility of the package by having the “data” object be a basic data frame and the “dataMetrics” object be a basic list of data frames. All of our examples ended up being for RNA-sequencing and our package is available on Bioconductor, and so we agree having the possibility for input as “SummarizedExperiment” object can be very useful.

The package now allows users to use the “SummarizedExperiment” input object (“dataSE”) as input. The assay() of the “dataSE” object is similar to the original “data” object (i.e. count table of reads) and the rowData() of the “dataSE” object is similar to the original “dataMetrics” object (i.e. significance metrics from edgeR etc.). As a result, users can now use “dataSE” instead of the combination of “data” and “dataMetrics”.

We have now:

1) Changed the input data type to allow SummarizedExperiment (“dataSE”) for all bigPint plots.

2) Provided an article describing how to create SummarizedExperiment input (https://lindsayrutter.github.io/bigPint/articles/summarizedExperiment.html)

3) Provided reproducible code to recreate all plots in our articles using the SummarizedExperiment input (instead of the data and dataMetrics input). The SummarizedExperiment versions are are located at the bottom of each article (https://lindsayrutter.github.io/bigPint/articles/static.html, https://lindsayrutter.github.io/bigPint/articles/interactive.html, https://lindsayrutter.github.io/bigPint/articles/clusters.html, and https://lindsayrutter.github.io/bigPint/articles/pipeline.html).

4) Updated help functions for all interactive and static bigPint plots to also provide reproducible example code for SummarizedExperiment format (https://lindsayrutter.github.io/bigPint/reference/index.html).

5) Introduced two corresponding example datasets having the SummarizedExperiment format (se_soybean_cn_sub and se_soybean_ir_sub) (https://lindsayrutter.github.io/bigPint/reference/index.html).

6) Created two functions that help with specific manipulations sometimes needed for SummarizedExperiment input (convertSEPair() and convertSESubsetGenes()) (https://lindsayrutter.github.io/bigPint/reference/index.html).

7) Updated our explanation of these input formats in our paper (Page 3 under “Basic input” subtitle)

Currently, we think it is best to have both options available for users (“data” and “dataMetrics” OR “dataSE”). This is because we have already peer-review published examples using “data” and “dataMetrics” and users who are using these formats.

5. How does bigPint scale? Have you tried analyzing single cell RNA-seq datasets?

Thank you for asking about scalability. We did not provide example analysis of single cell RNA-seq datasets because we believe most of bigPint plots are more suitable for bulk RNA-seq data. We did write a short segment about bigPint scalability in our previous methods paper (https://bmcbioinformatics.biomedcentral.com/articles/10.1186/s12859-019-2968-1) under the subsection “Plot scalability”.

Errors:

1. The example interactive application for the volcano plot is broken.

Thank you for catching this. We corrected the link and it should be working now (https://lindsayrutter.github.io/bigPint/articles/interactive.html).

2. None of the parallel coordinate plots show the correct labels for genes when hovering over the lines associated with genes.

Thank you for catching this problem. It is likely we fixed this issue. We just checked and it now seems that the correct labels are shown for genes when hovering over lines in the parallel coordinate plots.

An example is on (https://lindsayrutter.github.io/bigPint/articles/static.html#static-parallel-coordinates). There are ten genes overlaid as purple lines on the parallel coordinate plot. The minimum code to reproduce this plot is as follows:

library(bigPint)

library(dplyr)

data("soybean_ir_sub")

data("soybean_ir_sub_metrics")

soybean_ir_sub[,-1] <- log(soybean_ir_sub[,-1] + 1)

geneList = soybean_ir_sub_metrics[["N_P"]][1:10,]$ID

ret <- plotPCP(data = soybean_ir_sub, geneList = geneList, lineSize = 0.4, lineColor = "purple", saveFile = FALSE, hover = TRUE)

ret[[1]]

The read count table for these ten genes can be viewed with this code:

filter(soybean_ir_sub, ID %in% geneList)

It seems the hovered gene labels are correct. For instance, the lowest N.2 valued gene is Glyma.19G168700.Wm82.a2.v1 upon hovering and it has the lowest N.2 value in the read count table (1.098612). Similarly, the highest N.1 value gene is Glyma.11G044800.Wm82.a2.v1 upon hovering and it has the highest N.1 value in the read count table (7.447168).

If you do still see the problem, please let us know. Otherwise, it may be something that we have corrected.

**Reviewer #2:**

This is supplied as a R-markdown to show what I have done to try to use bigPint.

I have also supplied the file as an attachment.

---

title: "review_bigPint"

author: "Paul Brennan"

date: "12/10/2019"

output: html_document

---

\### Novelty in visualisation

It would be useful to see the package integrated into a workflow.

Thank you for this suggestion. We agree a workflow is crucial for new users. As we elaborate more below, we have a reproducible pipeline workflow with sample data. We now point to it much more clearly in the manuscript under a new section “Quick start”.

\### Minor concerns

I have some minor concerns that I believe need to be addressed prior to publication.

My first an most important concern is that I have found it quite difficult to reproduce the workflows and examples using the links in the manuscipt.

We originally structured the manuscript to highlight that our package can be used by both (1) biologists analyzing RNA-seq data, and (2) developers who would like to repurpose our double-layered activity for other uses. This review has been very insightful for us to realize that the original structure of our manuscript would have likely inadvertently caused readers in the first group (the biologists who simply want to apply the package toward their RNA-seq data) to fall into a very frustrating situation that could very well have caused them to never try our package. After reading this review, we see that you accessed some of the scripts that are only intended for developers to tweak for their own uses (or curious developers who want to understand the fine details of our code). We also see that it was unclear for you to know where to start hands-on using the package. Because of this review, we realize that (1) it was not helpful for us to include links to code for developers in our manuscript as they could easily be misunderstood as code that can be directly applied to analyze data, and (2) we did not suitably point biologists to a “quick start” location where they could begin testing the package quickly and determine whether it works for them. We have significantly restructured our manuscript to hopefully make it more clear where biologists can quickly start to apply our graphics. We also moved all detailed code intended for developers to the supplementary section to prevent sending biologists down a rabbit hole! Our changes will be explained throughout this review.

I have focussed my efforts on trying to make the bigPint package work using the links provided in the manuscript. Sadly, I have found that quite challenging.

For some reason, and it may be a limitation of my technical skills, when I accessed the code shown in the links for Table 1, I could not make the code work.

No, this was not a limitation of technical skills. This was unclear explanation on our part. We originally linked to the raw code (intended for developers to tweak) in Table 1. This code cannot typically be run directly by itself. We are really thankful to have this feedback because we immediately realized we should really only provide developer code in a supplementary section to not confuse biologists applying our code.

As a result, we removed the developer code from Table 1 and placed it in a supplementary file. Now Table 1 only has user-friendly resources to better understand bigPint graphics. We added a few sentences before Table 1 to indicate the separate access points for developers and biologists as follows:

“The current paper can benefit biology researchers and developers alike. Developers who would like to modify our code can access pseudocode and documented code separately in the supplementary material (see S1 Table). For biology researchers who would like to readily apply our visualization tools to their data, didactic materials (figures, applications, and videos) are available for each plotting type in Table 1.”

The first link to https://github.com/lindsayrutter/bigPint/blob/master/inst/shiny-examples/plotSMApp/app.R points to a very complex example. I do not believe it constitutes a "Helpful resource". When I copy and paste this data into a new R-Studio Project, it is very difficult to get to work.

We agree it is not a helpful resource, except for developers. To avoid confusion, as stated above, that link is now removed from the main manuscript and is only provided in the supplementary material.

In the supplementary material (S1 Table), we also provide a sentence to make it clear that it is intended for developers as follows: “The pseudocode and code provided in supplementary material is intended for developers who wish to understand the details of how we created the double-layered interactivity and possibly repurpose and modify these fairly new technology for new software. Biology researchers who wish to use the bigPint package should instead consult the bigPint package website (https://lindsayrutter.github.io/bigPint) and the associated manuscripts.”

\#### Trying to get Scatterplot Matrix App to work...

Here I am going to use the R Markdown file to catalogue my attempts.

Script from [here](bit.ly/spmCode)

```{r cut_and_paste_from_bit_ly_spmCode, include=FALSE, eval=FALSE}

\# I have removed this from the review because it uses up too many words...

\# trust me please, I have cut and pasted it verbatim.

```

When I run this, a window opens and then closes again. Interesting my data

is a Value - not Data in my Global Environment and it says "NULL (empty)"

Why? Well let's run the data acquisition by itself.

Annoyingly this is not a reproducible problem.

Sometimes it seems to work but I don't know why.

```{r test_data_download, include=TRUE}

data <- bigPint:::PKGENVIR$DATA

str(data)

```

Still NULL, sadly.

All the other code examples use the same code to get data in so I'm not going to try them here. I have tried them previously.

We apologize for this frustrating experience. Again, the link to this code has been moved to the supplemental section and emphasized for only developers.

\#### Where else can I go for help?

Let's check (Bioconductor)[https://www.bioconductor.org/packages/release/bioc/html/bigPint.html].

The page is a good starting point.

It has a green build which is a good sign.

Check out the (R-script)[https://www.bioconductor.org/packages/release/bioc/vignettes/bigPint/inst/doc/bioconductor.R]

Woops! It seems blank. A bit frustrating.

[HTML Vignette](https://www.bioconductor.org/packages/release/bioc/vignettes/bigPint/inst/doc/bioconductor.html)

points to website with a [rotten url](https://lindsayrutter.github.%20io/bigPint/)! Woops again!

We apologize for this inconvenience. We were informed about this broken link in an automatic message and have updated the broken link.

Let's go to the Github Repository and see what we can find...

The [link](https://lindsayrutter.github.io/bigPint/) is in the manuscript

and works. Yeah!!

OK, now we have some different code:

```{r test_data_download, include=TRUE}

library(bigPint)

data("soybean_cn_sub")

soybean_cn_sub <- soybean_cn_sub[,1:7]

app <- plotSMApp(data=soybean_cn_sub)

if (interactive()) {

shiny::runApp(app)

}

```

Good new is that the data() function works and gives us some data!

```{r str_data}

str(soybean_cn_sub)

```

Nice data.frame produced.

Bad news is that my interactive plot still won't work!

Well actually it does work when I run this again.

It is a bit slow and it gives lots of red text:

'scatter' objects don't have these attributes: 't2'

What happens if I try the

```{r try_PKGENVIR$DATA_again}

data <- bigPint:::PKGENVIR$DATA

```

It works! If I do it after I run your App. Wonder why? I'm not going to spend any time working through that at the moment.

Now the script above works with lots of error messages.

Thank you for pointing out these red text. We explain them later in this review.

\#### Back to square one, can I make the static hex plots

```{r static_hex}

data(soybean_ir_sub)

soybean_ir_sub[,-1] <- log(soybean_ir_sub[,-1]+1)

data(soybean_ir_sub_metrics)

ret <- plotLitre(data = soybean_ir_sub,

dataMetrics = soybean_ir_sub_metrics, threshVal = 1e-10,

saveFile = FALSE)

length(ret)

names(ret)[1]

ret[[1]]

```

Answer is Yes! Good and it looks interesting with points highlighted on it.

I probably should have started here in the first place but hey...

This is a very helpful point and we realize that we did not successfully point users to the best starting point. We have added a “Quick start” subsection to our manuscript that directs users to the reproducible pipeline on our website. This should hopefully help prevent users from scrambling around trying to find the working code. Specifically, we now state:

“For users who would like to immediately try out the package hands-on and apply bigPint graphics to their data, we recommend consulting the example pipeline (https://lindsayrutter.github.io/bigPint/articles/pipeline). This pipeline uses reproducible code and sample data from the bigPint package, so you can smoothly follow along each line of example code. For additional details, we recommend users to view articles in the Get Started tab on the package website (https://lindsayrutter.github.io/bigPint).”

\#### I need to try to make the apps work...

I found some code here: [https://rdrr.io/bioc/bigPint/man/plotVolcanoApp.html]

```{r plot_Volcano_App}

library(bigPint)

\# Example 1: Create interactive volcano plot of logged data using hexagon

\# bins for the background.

data(soybean_cn_sub)

data(soybean_cn_sub_metrics)

app <- plotVolcanoApp(data = soybean_cn_sub,

dataMetrics = soybean_cn_sub_metrics)

if (interactive()) {

shiny::runApp(app)

}

It seems our code gets automatically copied to various websites that we did not directly upload it to (including the https://rdrr.io/bioc/bigPint website). We changed our manuscript to help guide users to a better starting point (the reproducible pipeline and our website). This is in the new “Quick start” subsection of our manuscript.

```

OK, so this work. Excellent! I am reassured and in fact more than reassured.

I am pleased.

I can plot the gene subset and Download selected genes. I was under the impression that I would be able to click on a hexagon but I don't seem to be able to do that. Seems that functionality is not possible here.

Good point. Clicking on a background hexagon in the interactive volcano plot does not have functionality. That functionality is only available in the background hexagons of the scatterplot matrix, where points/genes within that clicked hexagon are then overlaid. The overlaid points are not necessarily interesting within the *same* scatterplot from which the hexagon was clicked (because all the points will just be directly overlaid onto the clicked hexagon itself). However, it is interesting to see how those points/genes are overlaid across all the other scatterplots within the scatterplot matrix.

In contrast, having the functionality to allow users to click on the background hexagon in the interactive volcano plot would not be of much interest because it would simply overlay the genes/points on top of that same clicked hexagon.

There are these subtle differences between the plotting types, so we list them in Table 2. Each plotting type had different challenges developing technicality, so Table 2 can help (1) biology researchers be less surprised/concerned if the interactive plot does not function as they might suspect and (2) developers identify certain forms of double-layer interactivity that could be useful to repurpose for other interactive software and know which plotting types may have that specific type of technique.

```{r plot_Volcano_App}

\# Example 2: Create interactive volcano plot of logged data using points for

\# the background.

app <- plotVolcanoApp(data = soybean_cn_sub,

dataMetrics = soybean_cn_sub_metrics, option = "allPoints",

pointColor = "magenta")

if (interactive()) {

shiny::runApp(app)

}

```

This shows the points. The scale of the P-value seems a little unwise.

Should it really go all the way up to 1? I think there should be the option

to go down to lower P-values more easily and not up.

Thank you for the helpful suggestion. After this comment, we realized that the slider input in Shiny may have less resolution than something like a text input. We updated the p-value user input to be in text format, so the user can now enter text to specify their p-values and have access to finer resolutions and very low values (such as 0.01, 0.00001, 5e-10, etc). An example of the new version can be viewed here: https://bigpint.shinyapps.io/volcano/.

\#### try PlotLitre App from online

```{r plot_Volcano_App}

data(soybean_ir_sub)

data(soybean_ir_sub_metrics)

soybean_ir_sub_log <- soybean_ir_sub

soybean_ir_sub_log[,-1] <- log(soybean_ir_sub[,-1]+1)

app <- plotLitreApp(data = soybean_ir_sub_log,

dataMetrics = soybean_ir_sub_metrics)

if (interactive()) {

shiny::runApp(app, port = 1234, launch.browser = TRUE)

}

```

I can see the number of genes in each hexagram if I hover over it - nice.

Plot gene works to give orange spots which can be hovered over to identify. However, I'm not sure how I am selecting those genes.

I have worked out that it is going through the genes by rank. However, because that information is off the bottom of the screen, it took me a while to realise.

I need to watch the video again!

In addition to the video, the “About” tab in the application can help users identify that the genes are plotted by gene rank.

Back to (website)[https://lindsayrutter.github.io/bigPint/articles/interactive.html]

Watch video about Scatterplot matrix app

Cut and paste code:

```{r plotSMAapp_again}

library(bigPint)

data("soybean_cn_sub")

soybean_cn_sub <- soybean_cn_sub[,1:7]

app <- plotSMApp(data=soybean_cn_sub)

if (interactive()) {

shiny::runApp(app)

}

```

This works! Excellent.

Selecting hexagrams works.

Downloading IDs works.

Downloading plots doesn't :-(

Need to open in Browser as advised!

Could add that as error message?

Thank you for this feedback. We are glad that our statement about this in the “About” tab (“ You need to view this application in a web browser for this function to work”) helped in this case. We considered adding this as an error message. However, for some reason, it seems that the plots can now download without being on a Browser. This may be an update with Plotly, other packages, RStudio version, etc. We added our sessionInfo() at the end of this review. It may not be necessary then for us to add this as an error message (and we may eventually remove the statement in the “About” tab).

OK so it does work but it produces LOTS of warnings.

I wonder why?

Warning: 'scatter' objects don't have these attributes: 't2'

Lots of repeat of this warning.

Thank you for asking about these warnings. The warning you mentioned about ‘scatter’ objects not having attributes ‘t2’ comes from a necessary user-interaction modification we added to the scatterplot matrix plotly object. By default, the plotly object outputs a verbose and non-informative format like (counts: 40, hexID = 143) when the user hovers over a given hexagon. We write a second text format (’t2’) for each hexagon so that it only output a format like (counts: 40) as the hexID object is meaningless to the user (although it is kept in the background code and fed into htmlwidgets to overlay the gene points when users click on the hexagons). In this case, the plotly object does not recognize scatter objects as having this attribute ‘t2’ and outputs this warning. These warnings come from necessary modifications we developed to allow for the double-layer interactivity. They do not indicate any underlying problems that the users should be concerned about, but indeed, the “warnings” could lead users to feel concerned. As a result, we added one line to suppress these warning options.

\#### Try plotLitreApp

```{r plotSMAapp_again}

data("soybean_ir_sub")

data("soybean_ir_sub_metrics")

soybean_ir_sub_log <- soybean_ir_sub

soybean_ir_sub_log[,-1] <- log(soybean_ir_sub[,-1]+1)

app <- plotLitreApp(data=soybean_ir_sub_log,

dataMetrics = soybean_ir_sub_metrics)

if (interactive()) {

shiny::runApp(app, port = 1234, launch.browser = TRUE)

}

```

\#### Try plotPCPApp

```{r plotPCPApp}

soybean_ir_sub_st = as.data.frame(t(apply(as.matrix(soybean_ir_sub[,-1]), 1,

scale)))

soybean_ir_sub_st$ID = as.character(soybean_ir_sub$ID)

soybean_ir_sub_st = soybean_ir_sub_st[,c(length(soybean_ir_sub_st),

1:length(soybean_ir_sub_st)-1)]

colnames(soybean_ir_sub_st) = colnames(soybean_ir_sub)

nID = which(is.nan(soybean_ir_sub_st[,2]))

soybean_ir_sub_st[nID,2:length(soybean_ir_sub_st)] = 0

plotGenes = filter(soybean_ir_sub_metrics[["N_P"]], FDR < 0.01, logFC < -4) %>%

select(ID)

pcpDat = filter(soybean_ir_sub_st, ID %in% plotGenes[,1])

app <- plotPCPApp(data = pcpDat)

if (interactive()) {

shiny::runApp(app, display.mode = "normal")

}

```

Works!

In Browser, I can save images as advised.

Nice job.

\#### Try Volcano app from website

Sadly no code on [website](https://lindsayrutter.github.io/bigPint/articles/interactive.html#volcano-plot-app) when accessed on 12 Oct 2019.

Thank you for pointing this out and sorry for the inconvenience. We added the interactive volcano plot code to the link.

Example code from (here)[https://rdrr.io/bioc/bigPint/man/plotVolcanoApp.html] instead as above. Made it work again!

GREAT!

```{r session_info}

sessionInfo()

```

So, I made it work but not from code on the manuscript.

Please make it easier for me.

Thank you for your helpful feedback here. We hope to avoid the unnecessary pains you experienced here from happening a second time to anyone else to reads our document. The main issue was us not clearly providing a quick starting point for users and a clear separation of what is intended for users versus developers. We believe that our updates (including a Quick Start section in our manuscript and separating developer-intended code into the supplemental section) will clear up what would have caused much confusion.

Here is my session info:

R version 3.6.1 (2019-07-05)

Platform: x86_64-apple-darwin15.6.0 (64-bit)

Running under: macOS Sierra 10.12.6

Matrix products: default

BLAS: /System/Library/Frameworks/Accelerate.framework/Versions/A/Frameworks/vecLib.framework/Versions/A/libBLAS.dylib

LAPACK: /Library/Frameworks/R.framework/Versions/3.6/Resources/lib/libRlapack.dylib

Random number generation:

RNG: Mersenne-Twister

Normal: Inversion

Sample: Rounding

locale:

[1] en_GB.UTF-8/en_GB.UTF-8/en_GB.UTF-8/C/en_GB.UTF-8/en_GB.UTF-8

attached base packages:

[1] stats graphics grDevices utils datasets methods

[7] base

other attached packages:

[1] shinycssloaders_0.2.0 Hmisc_4.2-0 Formula_1.2-3

[4] survival_2.44-1.1 lattice_0.20-38 RColorBrewer_1.1-2

[7] GGally_1.4.0 data.table_1.12.2 dplyr_0.8.3

[10] stringr_1.4.0 hexbin_1.27.3 tidyr_1.0.0

[13] htmlwidgets_1.5.1 plotly_4.9.0 ggplot2_3.2.1

[16] shinydashboard_0.7.1 shiny_1.4.0 bigPint_1.0.0

loaded via a namespace (and not attached):

[1] Rcpp_1.0.2 assertthat_0.2.1 zeallot_0.1.0

[4] digest_0.6.21 mime_0.7 R6_2.4.0

[7] plyr_1.8.4 backports_1.1.5 acepack_1.4.1

[10] httr_1.4.1 pillar_1.4.2 rlang_0.4.0

[13] lazyeval_0.2.2 rstudioapi_0.10 rpart_4.1-15

[16] Matrix_1.2-17 checkmate_1.9.4 labeling_0.3

[19] splines_3.6.1 foreign_0.8-72 munsell_0.5.0

[22] compiler_3.6.1 httpuv_1.5.2 xfun_0.10

[25] pkgconfig_2.0.3 base64enc_0.1-3 htmltools_0.4.0

[28] nnet_7.3-12 tidyselect_0.2.5 tibble_2.1.3

[31] gridExtra_2.3 htmlTable_1.13.2 reshape_0.8.8

[34] viridisLite_0.3.0 withr_2.1.2 crayon_1.3.4

[37] later_1.0.0 grid_3.6.1 jsonlite_1.6

[40] xtable_1.8-4 gtable_0.3.0 lifecycle_0.1.0

[43] magrittr_1.5 scales_1.0.0 stringi_1.4.3

[46] promises_1.1.0 latticeExtra_0.6-28 ellipsis_0.3.0

[49] vctrs_0.2.0 tools_3.6.1 glue_1.3.1

[52] purrr_0.3.2 crosstalk_1.0.0 fastmap_1.0.1

[55] yaml_2.2.0 colorspace_1.4-1 cluster_2.1.0

[58] knitr_1.25

Our session info is as follows:

R version 3.6.2 (2019-12-12)

Platform: x86_64-apple-darwin15.6.0 (64-bit)

Running under: macOS Catalina 10.15.1

Matrix products: default

BLAS: /System/Library/Frameworks/Accelerate.framework/Versions/A/Frameworks/vecLib.framework/Versions/A/libBLAS.dylib

LAPACK: /Library/Frameworks/R.framework/Versions/3.6/Resources/lib/libRlapack.dylib

locale:

[1] en_US.UTF-8/en_US.UTF-8/en_US.UTF-8/C/en_US.UTF-8/en_US.UTF-8

attached base packages:

[1] stats	graphics grDevices utils	datasets methods	base

other attached packages:

[1] shinylogs_0.1.7	shinycssloaders_0.3 Hmisc_4.3-1

[4] Formula_1.2-3	survival_3.1-8	lattice_0.20-38

[7] RColorBrewer_1.1-2	GGally_1.4.0	data.table_1.12.8

[10] dplyr_0.8.4	stringr_1.4.0	hexbin_1.28.1

[13] tidyr_1.0.2	htmlwidgets_1.5.1	plotly_4.9.2

[16] ggplot2_3.2.1	shinydashboard_0.7.1 shiny_1.4.0

[19] bigPint_1.3.0

loaded via a namespace (and not attached):

[1] RcppCCTZ_0.2.6	bitops_1.0-6

[3] matrixStats_0.55.0	bit64_0.9-7

[5] httr_1.4.1	GenomeInfoDb_1.22.0

[7] tools_3.6.2	backports_1.1.5

[9] DT_0.12	R6_2.4.1

[11] rpart_4.1-15	DBI_1.1.0

[13] lazyeval_0.2.2	BiocGenerics_0.32.0

[15] colorspace_1.4-1	nnet_7.3-12

[17] withr_2.1.2	tidyselect_1.0.0

[19] gridExtra_2.3	bit_1.1-15.2

[21] compiler_3.6.2	Biobase_2.46.0

[23] htmlTable_1.13.3	nanotime_0.2.4

[25] DelayedArray_0.12.2	labeling_0.3

[27] scales_1.1.0	checkmate_2.0.0

[29] digest_0.6.24	foreign_0.8-75

[31] XVector_0.26.0	base64enc_0.1-3

[33] jpeg_0.1-8.1	pkgconfig_2.0.3

[35] htmltools_0.4.0	fastmap_1.0.1

[37] rlang_0.4.4	rstudioapi_0.11

[39] RSQLite_2.2.0	farver_2.0.3

[41] zoo_1.8-7	jsonlite_1.6.1

[43] crosstalk_1.0.0	BiocParallel_1.20.1

[45] acepack_1.4.1	RCurl_1.98-1.1

[47] magrittr_1.5	GenomeInfoDbData_1.2.2

[49] Matrix_1.2-18	Rcpp_1.0.3

[51] munsell_0.5.0	S4Vectors_0.24.3

[53] lifecycle_0.1.0	yaml_2.2.1

[55] stringi_1.4.6	SummarizedExperiment_1.16.1

[57] zlibbioc_1.32.0	plyr_1.8.5

[59] grid_3.6.2	blob_1.2.1

[61] parallel_3.6.2	promises_1.1.0

[63] crayon_1.3.4	splines_3.6.2

[65] knitr_1.28	anytime_0.3.7

[67] pillar_1.4.3	GenomicRanges_1.38.0

[69] stats4_3.6.2	glue_1.3.1

[71] latticeExtra_0.6-29	png_0.1-7

[73] vctrs_0.2.2	httpuv_1.5.2

[75] gtable_0.3.0	purrr_0.3.3

[77] reshape_0.8.8	assertthat_0.2.1

[79] xfun_0.12	mime_0.9

[81] xtable_1.8-4	later_1.0.0

[83] rsconnect_0.8.16	viridisLite_0.3.0

[85] tibble_2.1.3	memoise_1.1.0

[87] IRanges_2.20.2	cluster_2.1.0

[89] ellipsis_0.3.0
